# IL-15 Expression on RA Synovial Fibroblasts Promotes B Cell Survival

**DOI:** 10.1371/journal.pone.0040620

**Published:** 2012-07-09

**Authors:** Marta Benito-Miguel, Yolanda García-Carmona, Alejandro Balsa, María-Belén Bautista-Caro, Irene Arroyo-Villa, Tatiana Cobo-Ibáñez, María Gema Bonilla-Hernán, Carlos Pérez de Ayala, Paloma Sánchez-Mateos, Emilio Martín-Mola, María-Eugenia Miranda-Carús

**Affiliations:** 1 Department of Rheumatology, Hospital Universitario La Paz, Madrid, Spain; 2 Laboratorio de Inmuno-oncología, Hospital General Universitario Gregorio Marañón Madrid, Spain; University Hospital Jena, Germany

## Abstract

**Introduction:**

The purpose of this study was to examine the role of RA Synovial Fibroblast (RASFib) IL-15 expression on B cell survival.

**Methods:**

Magnetically sorted peripheral blood memory B cells from 15 healthy subjects were cocultured with RASFib.

**Results:**

RASFib constitutively expressed membrane IL-15. Survival of isolated B cells cultured for 6 days, below 5%, was extended in coculture with RASFib to 52+/−8% (p<0.001). IL-15 neutralizing agents but not isotype controls, reduced this rate to 31+/−6% (p<0.05). Interestingly, rhIL-15 had no effect on isolated B cells but significantly increased their survival in coculture with RASFib. In parallel, B cell IL-15R chains were upregulated in cocultures. BAFF and VCAM-1, that are expressed on RASFib, were tested as potential candidates involved in upregulating B cell IL-15R. Culture of B cells in the presence of rhBAFF or rhVCAM-1 resulted in significantly increased survival, together with upregulation of all three IL-15R chains; in parallel, rhIL-15 potentiated the anti-apoptotic effect of BAFF and VCAM-1. Both BAFF and VCAM-1 neutralizing agents downmodulated the effect of RASFib on B cell survival and IL-15R expression. In parallel, rhIL-15 had a lower effect on the survival of B cells cocultured with RASFib in the presence of BAFF or VCAM-1 neutralizing agents. Peripheral blood B cells from 15 early RA patients demonstrated an upregulated IL-15R and increased survival in cocultures.

**Conclusion:**

IL-15 expression on RASFib significantly contributes to the anti-apoptotic effect of RASFib on B cells. IL-15 action is facilitated by BAFF and VCAM-1 expressed on RASFib, through an upregulation of IL-15R chains.

## Introduction

The inflamed synovium of Rheumatoid Arthritis (RA) is characterized by a hyperplastic lining layer of macrophages and fibroblasts (RASFib) [1]. In addition, the adjacent sublining layer contains an infiltrate of myeloid and lymphoid cells that in most patients is diffuse, with immune cells randomly distributed among resident fibroblasts and endothelial cells [2]. Alternatively in some 20% of patients, T and B cells are arranged in defined follicles designated as aggregates and yet in rarer cases, infiltrating lymphoid cells form ectopic germinal centers [3].

B cells can contribute to the pathogenesis of RA synovitis through the local production of antibodies [4], chemokines and cytokines, and acting as efficient antigen presenting cells (APCs) [5]. The mechanisms leading to B cell accumulation in the RA synovium are not fully understood, and several reports have demonstrated a pivotal role of direct B cell/RASFib interactions [6]–[11]. In fact, infiltrating B lymphocytes and plasma cells have been observed in close contact with RASFib in the subintimal layer [6]. Furthermore, RASFib seem to have properties of FDCs [7], and express B cell trophic factors such as VCAM-1 [8]–[10] and BAFF [11]–[13]. In addition, IL-15 expression has been observed in the intimal and subintimal layer of the RA synovial membrane [14], is transiently upregulated in the synovial fluid of early RA patients [15], and we have reported that constitutively expressed IL-15 on the surface of RASFib is biologically active on cocultured T lymphocytes through direct cell contact [16], [17].

The cytokine IL-15 shares many properties with IL-2 [18] and acts through a heterotrimeric receptor consisting of a specific high-affinity binding α-chain (designated as IL-15Rα) plus the IL-2Rβ- and common γ-chain, that are responsible for signaling [19], [20]. Armitage et al first described in 1995 that IL-15 costimulates the proliferation and differentiation of activated B cells, but has no stimulatory effect on resting B cells [21], and it has more recently been reported that IL-15 on the surface of follicular dendritic cells enhances germinal center B cell proliferation [22]. Therefore, our objective was to examine the effect of RASFib IL-15 on peripheral blood B cells.

Circulating peripheral blood B cells from untreated, early RA patients are likely to be activated and display heightened responses when cocultured with RASFib. Our early arthritis clinic allowed the study of B cells from early RA patients who have not received disease modifying drugs (DMARDs) or steroids, thereby minimizing interference of drugs with in vitro B cell responses.

We observed that IL-15 expression on RASFib significantly promoted the survival of cocultured B cells. Interestingly, the action of IL-15 was facilitated by BAFF and VCAM-1 expressed on RASFib, through an upregulation of IL-15R chains. In addition, peripheral blood B cells from early RA patients demonstrated a constitutively upregulated expression of IL-15R chains together with and increased survival rate in cocultures.

## Methods

### Ethics Statement

The study was approved by the Hospital La Paz – IdiPAZ Ethics Committee, and all subjects provided written informed consent.

### Patients

Synovial membranes were obtained from 10 RA patients with established disease undergoing synovectomy or arthroplasty, and from 10 osteoarthritis (OA) patients undergoing arthroplasty (Table 1). Peripheral blood was obtained from 15 healthy controls and from 15 early RA patients fulfilling at least four American College of Rheumatology criteria [23], who had never received disease-modifying drugs or corticosteroids and with a disease duration of <6 months (Table 2). La Paz University Hospital in Madrid, Spain, has a monographic clinic that takes care of early arthritis patients referred from a wide primary care area. This facilitated recruitment of untreated early RA patients for the present study. Among early RA patients there were 3 male and 12 female, 13 (87%) tested positive for IgM rheumatoid factor, their ages were 23–76 years (mean 49.72, SD 15.52, median 46), duration of symptoms at first evaluation was from 2 to 24 wk (mean 9.23, SD 5.7, median 8.8), and disease activity score 28 (DAS28) [24] at first evaluation was from 4.95 to 7.76 (mean 5.85, SD 0.81, median 5.59) (Table 2). Eight patients with early RA donated blood for a second time, once disease activity had been controlled. These patients were receiving 15 mg of oral MTX weekly, except for one patient who was taking 20 mg per week. Additionally, 2 patients were taking prednisone, 2.5 mg daily. IgM rheumatoid factor was positive at initial evaluation in 7 of these 8 patients; there were 1 man and 7 women, with ages of 41.52±11.39 years (mean±SD). DAS28 before initiation of treatment was 5.92±0.77 (mean±SD) and at the time of the second blood drawing it was 1.91±0.4. These patients were in remission as defined by a DAS28 score <2.6 [25] (Table 2).

**Table 1:** Clinical data of patients providing surgical synovial samples.

	Established RA	OA
Number	10	10
Age (mean ± SD)	68+	70+
Gender (Male/Female)	2/8	3/7
Disease duration (mean ± SD)	17±6 years	12±5 years
Rheumatoid factor +	8 (80%)	0 (0%)
Anti-CCP +	7 (70%)	0 (0%)
DAS28 (mean ± SD)	3.6±0.6	N/A

**Table 2:** Clinical data of early RA patients at first evaluation and of early RA patients (8 out of 15) who donated blood for a second time, once remission had been achieved.

	Early RA (1^st^ evaluation)	Early RA (2^nd^ evaluation)
Number	15	8
Age (mean ± SD)	49.72±15.52 yrs	41.52±11.39
Gender (Male/Female)	3/12	1/7
Disease duration (mean ± SD)	9.23±5.7 weeks	16.2±1.5 months
Rheumatoid factor + (%)	13 (86.7%)	7 (87.5%)
Anti-CCP + (%)	11 (73%)	6 (75%)
DAS28 (mean ± SD)	5.85±0.81	1.91±0.4
Medication	NSAIDs	Oral MTX±PRD

### Culture of Human Fibroblasts

**Figure 1:**
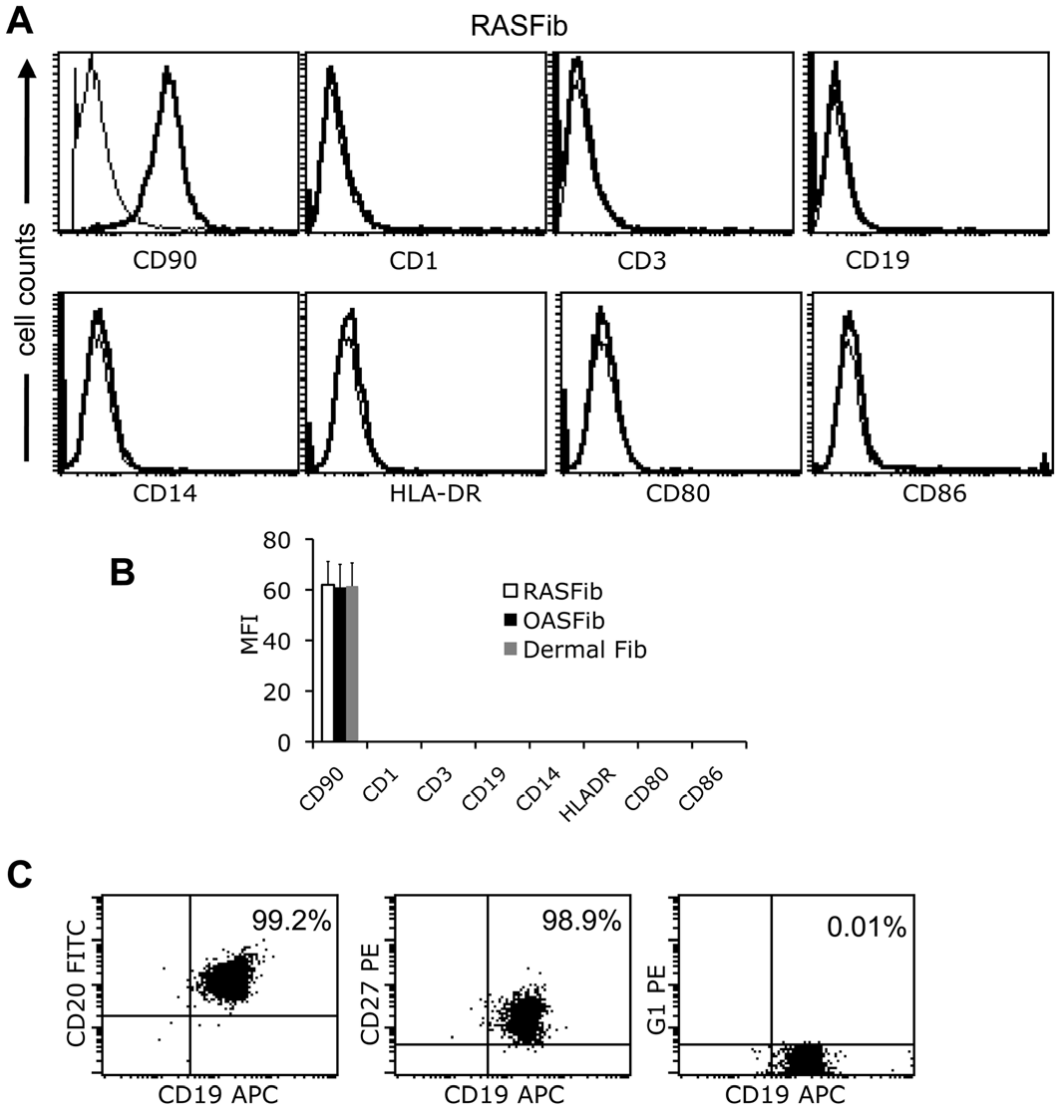
Characterization of cultured RASFib, OASFib and dermal fibroblasts (Third passage) and of freshly isolated memory B cells. **A**. Representative flow cytometry histograms show staining of cultured RASFib (Third passage) with anti-CD90, anti-CD1, anti-CD3, anti-CD19, anti-CD14, anti-HLA-DR, anti-CD80 and anti-CD86 (Thick lines) or appropriate isotype controls (Thin lines). **B**. Bar histogram showing the MFI of RASFib (n = 10 lines), OASfib (n = 10 lines) or dermal fibroblasts (n = 5 lines) on the thrid passage, after staining with the antibodies mentioned in A. **C**. Representative flow cytometry dot plots after triple staining of isolated memory B cells with anti-CD20 FITC, anti-CD19 APC and anti-CD27PE or isotype control (gamma1) PE. Shown are two-dimension dot plots with different combinations of the above mentioned fluorochrome-labeled antibodies.

RASFib and OASFib were obtained by collagenase digestion (type I; Worthington Biochemical, Freehold, NJ) of human synovial tissue obtained at arthroplasty or synovectomy. Dermal fibroblasts were obtained by collagenase digestion of normal skin obtained from punch biopsies of five healthy volunteers. Cells were plated in 75-cm2 flasks (Corning Life Sciences, Amsterdam, The Netherlands) and grown in DMEM (Lonza, Basel, Switzerland) supplemented with 10% FCS (Lonza), 2 mM L-glutamine, 50 U/ml penicillin, and 50 mg/ml streptomycin (Lonza). Cells were passaged at 1/2 dilution when reaching 95% confluence, by gentle trypsinization (0.05% trypsin/0.53 mM EDTA; Gibco-Invitrogen, Carlsbad, CA). Fibroblasts were used between the third and fifth passages. At this time, they appear to be a homogeneous population of fibroblast-like cells that stain positive with anti-Thy-1 (CD90) Ab [26] and are negative for the expression of CD1, CD3, CD19, CD14, HLA class II, CD80, and CD86, as determined by flow cytometry and fluorochrome-conjugated monoclonal antibodies (BD Pharmingen, San Jose, CA) (Figure 1A, B).

### Memory B Cell Purification

Peripheral blood mononuclear cells (PBMCs) were separated from the peripheral blood by Ficoll-Paque Plus (GE Healthcare, Uppsala, Sweden) density gradient centrifugation. Highly purified B cells were isolated from PBMCs by exhaustive immunomagnetic depletion in an Automacs (Miltenyi Biotec, Bergisch Gladbach, Germany) using the “B Cell Isolation Kit II” (Miltenyi Biotec), containing a cocktail of biotin-conjugated antibodies against CD2, CD14, CD16, CD36, CD43, CD235a (Glycophorin A), and anti-biotin microbeads. Purity of B cells as assessed by flow cytometry was >99% CD19+. Subsequently, memory B cells (CD19+CD27+) were positively selected using CD27+ microbeads (Miltenyi Biotec), and purity of CD19+CD20+CD27+ cells by flow cytometry was ≥98% (Figure 1C). Memory B cells were used immediately after isolation. Memory B cells will henceforth be referred to as “B cells”.

### Culture of Isolated B Cells

Isolated memory B cells were cultured in 96-well flat-bottom plates (Corning) (10^5^ cells/well) with RPMI 1640 medium (Lonza) containing 10% FCS, 2 mM L-glutamine, 50 U/ml penicillin, 50 µg/ml streptomycin and 50 µM 2-mercapto-ethanol (“complete RPMI medium”). In some conditions, medium was supplemented with recombinant human IL-15 (rhIL-15), 1 to 400 ng/ml (R&D Systems, Abingdon, U.K.) or with rhBAFF, 1 to 400 ng/ml (R&D Systems). For VCAM-1 stimulation experiments, flat-bottom 96-well plates were coated with 10 µg/ml rhVCAM-1 (R&D Systems) for 1 hour at 37°C; memory B cells were subsequently cultured on coated wells in complete RPMI 1640 medium.

### Coculture Conditions

Experiments were performed with confluent fibroblast cultures prepared three days before contact. All experimental conditions were performed in triplicate and variation between replicates was <5%. RASFib were seeded in 96-well flat-bottom plates at 5×10^3^ cells/well. Three days later, the separated memory B cells (1×10^5^ cells/well) were added in fresh complete RPMI medium. B cells were harvested after 24, 48, 72 h, 4, 5 and 6 days by thorough washing with cold, serum-free medium. In some experiments, rhIL-15 (50 ng/ml) was added right after initiating cocultures.

#### Transwell system

A 0.4- µm Transwell system (Corning) was used to conduct some coculture experiments. The system consists of two compartments: a top well, with a porous matrix (0.4 µm), and a bottom well. This setup allows coculture of two types of cells to grow in the same medium with soluble factors exchanged through the pores, while preventing direct contact between them. RASFib were grown to confluence in the bottom well, and B cells were added either to the same well, allowing contact, or in the top well, avoiding contact.

### Assessment of Cell Viability

Cell viability was assessed by flow cytometry after staining with 10 µM JC-1 (5,5′,6,6′-tetrachloro-1,1′,3,3′-tetraethylbenzimidazol-carbo-cyanine iodide, Molecular Probes - Invitrogen, Leiden, The Netherlands) to evaluate the mitochondrial membrane potential or with annexin V/7AAD (7-Amino-actinomycin D) **(**BD PharMingen) to evaluate plasma membrane integrity, according to the manufacturers’ instructions.

### Neutralization Assays

Parallel experiments included the addition of blocking agents against IL-15, BAFF or VCAM-1: a neutralizing anti-IL-15 mAb (10 µg/ml; mab247, mouse IgG1; R&D Systems), a recombinant IL-15Rα/human Fcγ1 chimera (100 ng/ml; 147­IR, R&D Systems), an IL-15 mutant/murine Fcγ2a chimera, which is a high-affinity receptor site-specific antagonist for IL-15Rα [27], (10 µg/ml; HF 22015, Chimerigen, Allston, MA), an anti-BAFF mAb (5 µg/ml; mab124, mouse IgG2b, R&D systems) or an anti-VCAM-1 mAb (30 µg/ml; BBA6, mouse IgG1, R&D systems). In addition, the binding control anti-HLA class I (clone W6/32, 10 µg/ml; Sigma-Aldrich), or appropriate isotype controls (murine IgG1, murine IgG2a, murine IgG2b or human IgG1; all from R&D Systems) were used. These Abs were incubated with RASFib for 30 min at 37°C, and B cells were subsequently added without washing.

### Surface Cell Staining and Flow Cytometry

For surface IL-15 staining, RASFib were harvested on ice with PBS/5mM EDTA, washed with PBS/2% FCS/0.01% NaN_3_, and incubated on ice for 1 h with an anti-IL-15 mAb (mab 2471, R&D systems) or an irrelevant isotype control mAb. After further washing with PBS/2% FCS/0.01% NaN3, cells were incubated with an Alexa Fluor 488-goat anti-mouse Ab, washed once with PBS/2%FCS/0.01% NaN3 and once with PBS. Cells were then resuspended in 1% paraformaldehyde and analyzed in a FACSCalibur flow cytometer using CellQuest software (BD Biosciences). Surface IL-15Rα and surface BAFF were detected with an anti-IL-15Rα mAb (R&D Systems) or an anti-BAFF mAb (both from R&D systems), followed by an Alexa Fluor-488 or an Alexa Fluor-647 goat anti-mouse affinity purfied Ab (both from Molecular Probes/Invitrogen ). VCAM-1, IL-15 receptor β, and common γ chain were detected with PE-labeled anti-VCAM-1, PE-labeled anti-IL-2/IL-15Rβ (CD122), or PE-labeled anti-common γ chain (CD132) versus a PE-labeled isotype-matched control mAb (all from BD PharMingen, San José, CA). Fluorochrome-conjugated mAbs from BD PharMingen were used to examine the expression of the phenotypic markers CD19, CD20, CD27, CD1, CD3, CD14, HLA DR, CD80, and CD86. Mean fluorescence intensity (MFI) values are given as the difference between the MFI of tested cells and the MFI of background staining. Fold of induction for IL-15 receptor chains was calculated as “MFI of cocultured or treated cells/MFI freshly isolated cells”.

### Soluble Cytokine Detection

ELISAs for BAFF and IL-15 were performed in cell-free supernatants using kits from R&D Systems, Abingdon, UK, following the manufacturer’s instructions.

**Figure 2:**
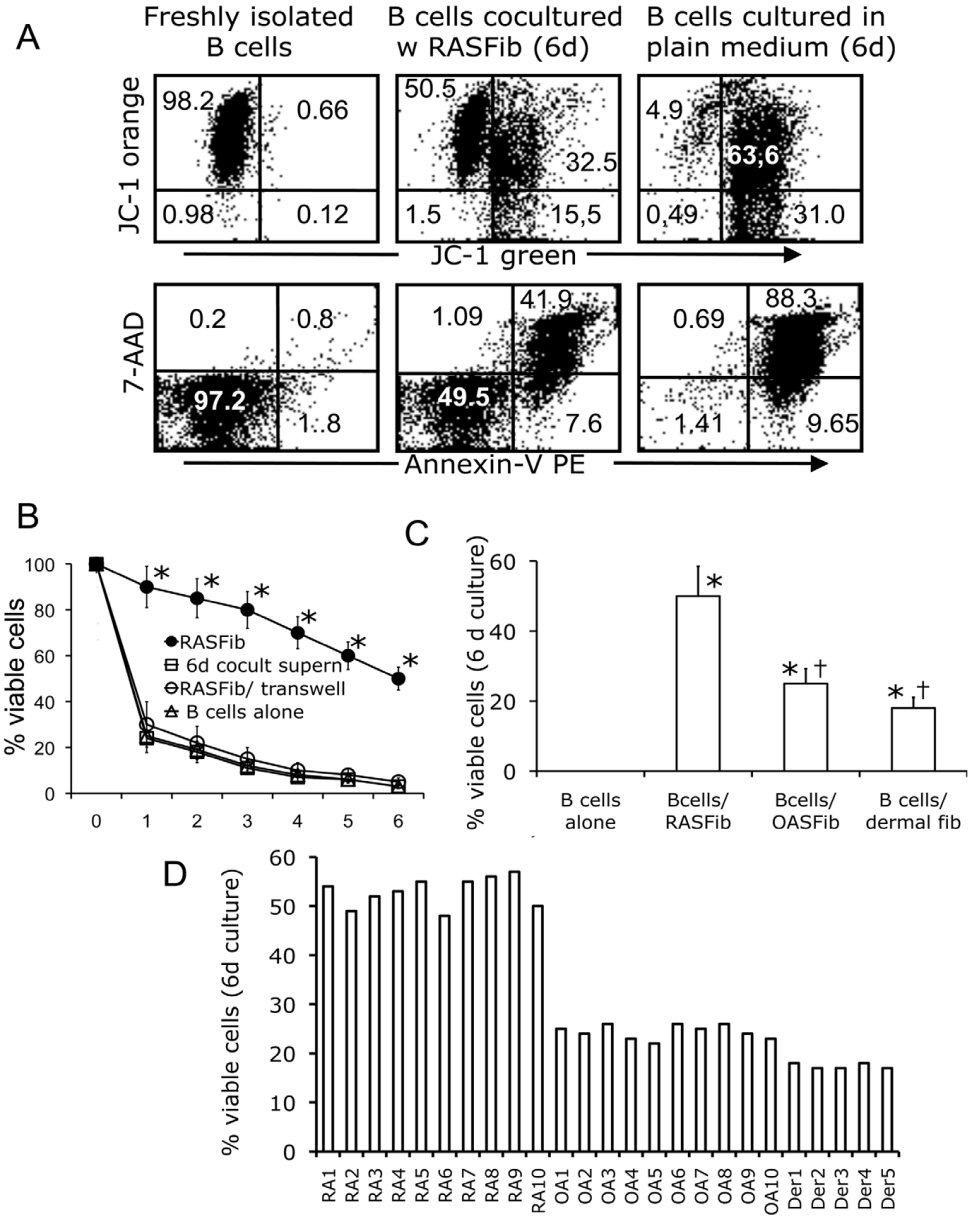
Effect of RASFib on B cell survival. Coculture with RASFib significantly increases B cell survival in a cell-contact dependent manner. **A**. Representative flow cytometry dot-plots of B cells stained with JC-1 or with 7AAD/Annexin V. When compared with freshly isolated B cells, viability of B cells cultured in plain medium for 6 days is dramatically decreased. Coculture with RASFib for 6 days significantly improves B cell survival. **B**. Percentage of viable B cells at different time points (0 to 6 days) (JC-1 staining). B cells were cultured in plain medium or cocultured with untreated RASFib, with RASFib and transwell inserts, or cultured with supernatants from 6-day RASFib/Bcell cocultures. Each point represents the mean and SD of 15 subjects. * p<0.05 vs B cells cultured in plain medium. **C**. RASFib are significantly more effective than OASFib or dermal fibroblasts at promoting B cell suvival. Shown is the percentage of viable cells present at 6 days cocultures of B cells with RASFib versus cocultures with OA or dermal fibroblasts, versus B cells cultured alone (JC-1 staining). Each bar represents the mean and SD of 15 subjects per group. *p<0.05 vs B cells cultured alone in plain medium; †p<0.05 vs B cells cocultured with RASFib. **D**. Variation of the effect of different RA, OA or dermal fibroblast cell lines on B cell survival was small. B cells from a single donor were cocultured for 6 days with 10 different RA synovial fibroblast lines (RA 1-10), 10 different OA synovial fibroblast lines (OA 1-10) or 5 different dermal fibroblast lines (Der 1–5). Shown is the percentage of viable B cells on the 6th day of coculture with each of the tested lines (JC-1 staining).

### Quantitative RT-PCR

For RNA extraction, B cells were collected as described above. Contamination by RASFib was ruled out by flow cytometry and by the absence of amplification of typical RASFib markers by RT-PCR, such as CD 90 (Thy-1). Total cellular RNA was isolated using the RNAQueous kit (Ambion-Applied Biosystems, Austin, TX) with DNAse treatment. For each sample, 1 µg of total RNA was subjected to reverse transcription using an Advantage RT for PCR kit (BD-Clontech, Palo Alto, CA), according to the manufactureŕs instructions. Aliquots (1 µl) of the reverse transcription products were used for quantitative PCR in the LightCycler™ PCR and Detection System (Roche Molecular Biochemicals, Mannheim, Germany) using the FastStart DNA Master SYBR Green I kit (Roche Diagnostics) as described by the manufacturer. The PCR reactions were set up in microcapillary tubes in a volume of 20 µl. The following sense and antisense primers were used: IL-15Rα primers are located in exon 2 (sense) and exon 5 (antisense) to amplify mRNA encoding all IL-15Rα variants that contain exon 2, and thus, bind IL-15: IL-15Rα sense 5′- GGA ATT CAT CAC GTG CCC TCC CCC CAT G -3′; IL-15Rα antisense, 5′- CGG GAT CCT CAA GTG GTG TCG CTG TGG CCC TG –3′ (product size, 543/444). IL-2/IL15Rβ sense, 5′- ACC TCT TGG GCA TCT GCA GC- 3′; IL-2/IL15Rβ antisense 5′- CGT CTC CAG GCA GAT CCA TT –3′ (product size, 531); common γ chain sense 5′- CCA GAA GTG CAG CCA CTA TC- 3′, common γ chain antisense 5′- TCA CTC CAA TGC TGA GCA CT –3′ (product size, 420). Quantitative PCR was performed in triplicate as previously described [15]. As an external standard, the transcript of 18S rRNA was amplified from the same cDNA samples using primers manufactured by Ambion. Quantities of specific mRNA in the sample were measured according to the corresponding gene-specific standard curve. The results are expressed as fold of induction: (cDNA sample cultured cells/18S cultured cells)/(cDNA sample freshly isolated cells/18S freshly isolated cells).

### Statistical Analysis

Comparison between groups was by Mann-Whitney U test. Paired samples were compared using a Wilcoxon matched pairs signed rank sum test. When appropriate, Bonferroni correction for multiple comparisons was applied.

## Results

### Effect of RASFib on B Cell Survival

The percentage of viable B cells obtained afer staining with JC-1 or annexinV/7AAD were comparable (Figure 2A), and results using JC-1 are shown henceforth throughout the manuscript. When compared with freshly isolated B cells (98,6±0.7% viability), viability of B cells cultured in plain medium dramatically decreased over time (3.6±1.9% viability on the 6^th^ day) (Figure 2A, B). Coculture with RASFib significantly improved B cell survival (53,2±8.1% viability on the 6^th^ day) (p<0.01) (Figure 2A, B). This effect was cell-contact dependent: when cocultures were established in the presence of transwell inserts that do not allow contact between B cells and fibroblasts, B cell survival was very short, as observed in isolated B cell cultures (Figure 2B), and significantly lower than survival observed when direct contact between B cells and fibroblasts was allowed (Figure 2B). Furthermore, the addition of supernatants from 6 day RASFib/B cell cocultures to isolated B cells did not increase survival (Figure 2B).

RASFib were significantly more effective than OASfib or dermal fibroblasts at promoting B cell suvival (Figure 2C), as indicated by the percentage of viable cells present at 6 days cocultures of B cells with RASFib versus cocultures with OA or dermal fibroblasts, versus B cells cultured alone. All of the tested RA synovial fibroblast lines had comparable effects on B cell survival (Figure 2D); likewise, variations among different OASFib lines and dermal fibroblast lines were also small when comparing their effects on B cell viability (Figure 2D).

### Effect of IL-15 Neutralizing Agents on the Survival of B Cells Cocultured with RASFib

**Figure 3:**
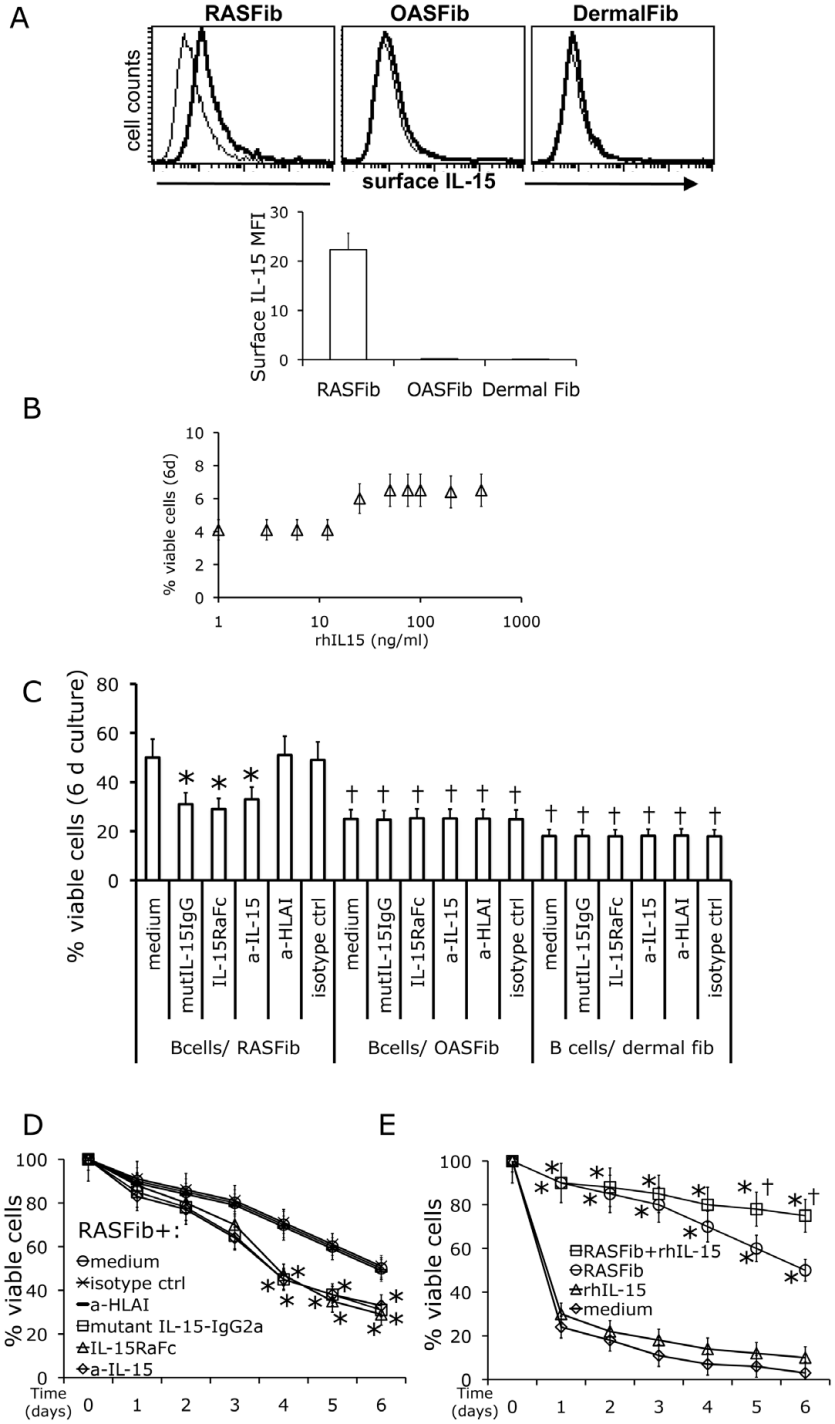
Effect of IL-15 neutralizing agents and of exogenous rhIL-15 on the survival of B cells cocultured with RASFib. The effect of RASFib on B cell survival is modified by IL-15 neutralizing agents and by exogenous rh IL-15. **A**. Flow cytometry of non-permeabilized cells demonstrates surface IL-15 expression on RASFib, OASFib and dermal fibroblasts. Top panels: representative flow cytometry histograms. Lower panel: bar histogram showing the mean and SD of the MFI (mean fluorescence intensity) of all tested RASFib cell lines (n = 10), OASFib lines (n = 10) and dermal fibroblast lines (n = 5). **B**. Effect of rhIL-15 (1–400 ng/ml) on isolated B cells cultured alone for 6 days. Each point represents the mean and SD of 15 subjects**. C**. Effect of IL-15 neutralizing agents on the survival of B cells cocultured with RASFib, OASFib or dermal fibroblasts (single time point, 6^th^ day of coculture). Each point represents the mean and SD of 15 subjects. * p<0.05 vs same condition in the absence of neutralizing agents. †p<0.05 vs B cells cocultured with RASFib in the absence of IL-15 neutralizing agents. **D**. Effect of IL-15 neutralizing agents on the survival of B cells cocultured with RASFib. Time-course (0–6 day coculture). Each point represents the mean and SD of 15 subjects. * p<0.05 vs conditions in the absence of neutralizing agents. **E**. rhIL15 has a minimal effect on the survival of B cells cultured alone but significantly upregulates the survival of B cells cocultured with RASFib. Each point represents the mean and SD of 15 subjects. *p<0.05 vs B cells cultured in plain medium. † p<0.05 vs B cells cultured with RASFib in the absence of rhIL-15.

As previously described [15], RASFib but not OASFib or dermal fibroblasts, constitutively expressed IL-15 on the cell membrane (Figure 3A). In contrast, the concentration of soluble IL-15 in cell-free supernatants was very low: 4.2±1.5 pg/ml. Of note, exogenous recombinant human IL-15 had a minimal effect on B cell survival at doses of 50 ng/ml and above and no effect at doses below 50 ng/ml ( = 50,000 pg/ml) (Figure 3B).

IL-15 neutralizing agents significantly reduced the survival of B cells cocultured with RASFib (Figure 3C,D). All of the three tested IL-15 neutralizing agents were effective: an antagonistic IL-15 mutant/Fcγ2a fusion protein that binds to the IL-15 receptor but does not induce signal transduction [25], an IL-15Rα-Fc fusion protein and a neutralizing anti-IL-15 mAb (Figure 3C,D). In contrast, B cell survival was not modified in the presence of isotype control agents or in the presence of the binding control anti-HLA class I (W6/32) (Figure 3C,D). Neutralization of IL-15 had no effect on the life span of B cells cocultured with OASFib or dermal fibroblasts (Figure 3C).

### Effect of RASFib on IL-15R Expression

Interestingly, whereas exogenous recombinant human IL-15 (1–400 ng/ml) had a minimal effect on B cell survival when added to isolated B cells in culture (Figure 3B), in the presence of RASFib, recombinant human IL-15 (50 ng/ml) significantly increased B cell survival above that observed in cocultures established in plain medium (Figure 3E).

**Figure 4:**
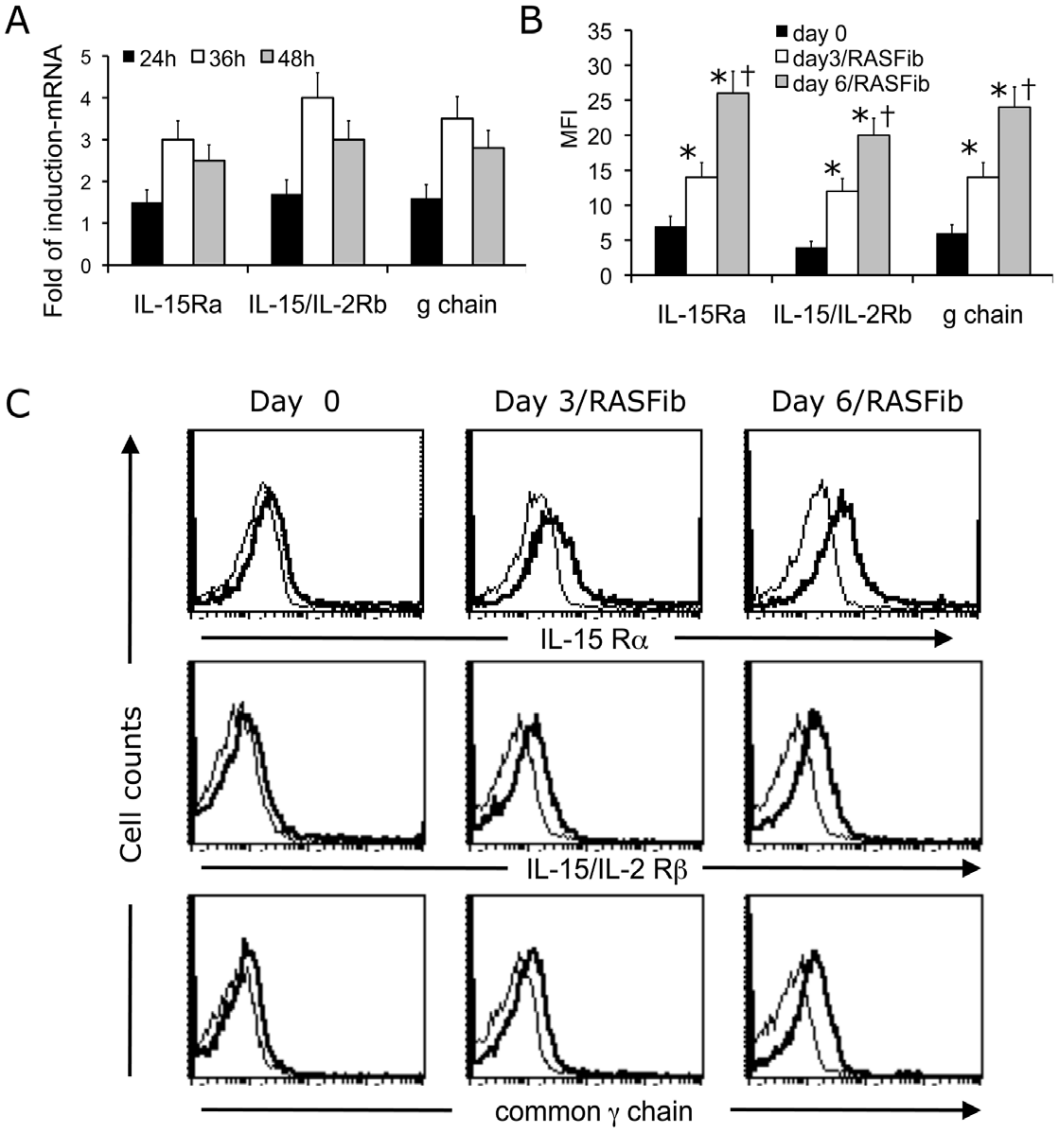
Effect of RASFib on the expression of IL15R α, β and γ chains on cocultured B cells. B cells cocultured with RASFib upregulate IL-15 α, β and γ chain expression. **A**. Real-time quantitative analysis of IL-15R mRNA expression in B cells cocultured with RASFib for 24, 36 or 48 h. Shown is fold of induction referred to expression of IL-15R α, β and γ chain in freshly isolated B cells. Each bar represents the mean and SD of 15 subjects. **B**. Expression of IL-15R α, β and γ chain on the surface of non-permeabilized B cells as determined by flow cytometry. Shown is expression on freshly isolated B cells, on B cells cocultured with RASFib for 3 days and on B cells cocultured with RASFib for 6 days. Each bar represents the mean and SD of 15 subjects. *p<0.05 vs freshly isolated B cells (day 0); † p<0.05 vs B cells cocultured for 3 dys with RASFib (day 3). **C**. Representative flow cytometry histograms showing the expression of IL-15R α, β and γ chains on freshly isolated B cells and on B cells cocultured with RASFib for 3 or 6 days.

Therefore we next sought to examine the expression of IL-15R on freshly isolated B cells and on B cells cocultured with RASFib (Figure 4). B cells cocultured with RASFib significantly upregulated IL-15 Rα, β and γ chain expression at the mRNA (Figure 4A) and protein levels (Figure 4 B, C). This indicates that RASFib facilitate the action of IL-15 on B cells through an upregulation of B cell IL-15R chains.

### Effect of BAFF and of VCAM-1 on B Cell Expression of IL-15R and on the Survival of B Cells Cocultured with RASFib

**Figure 5:**
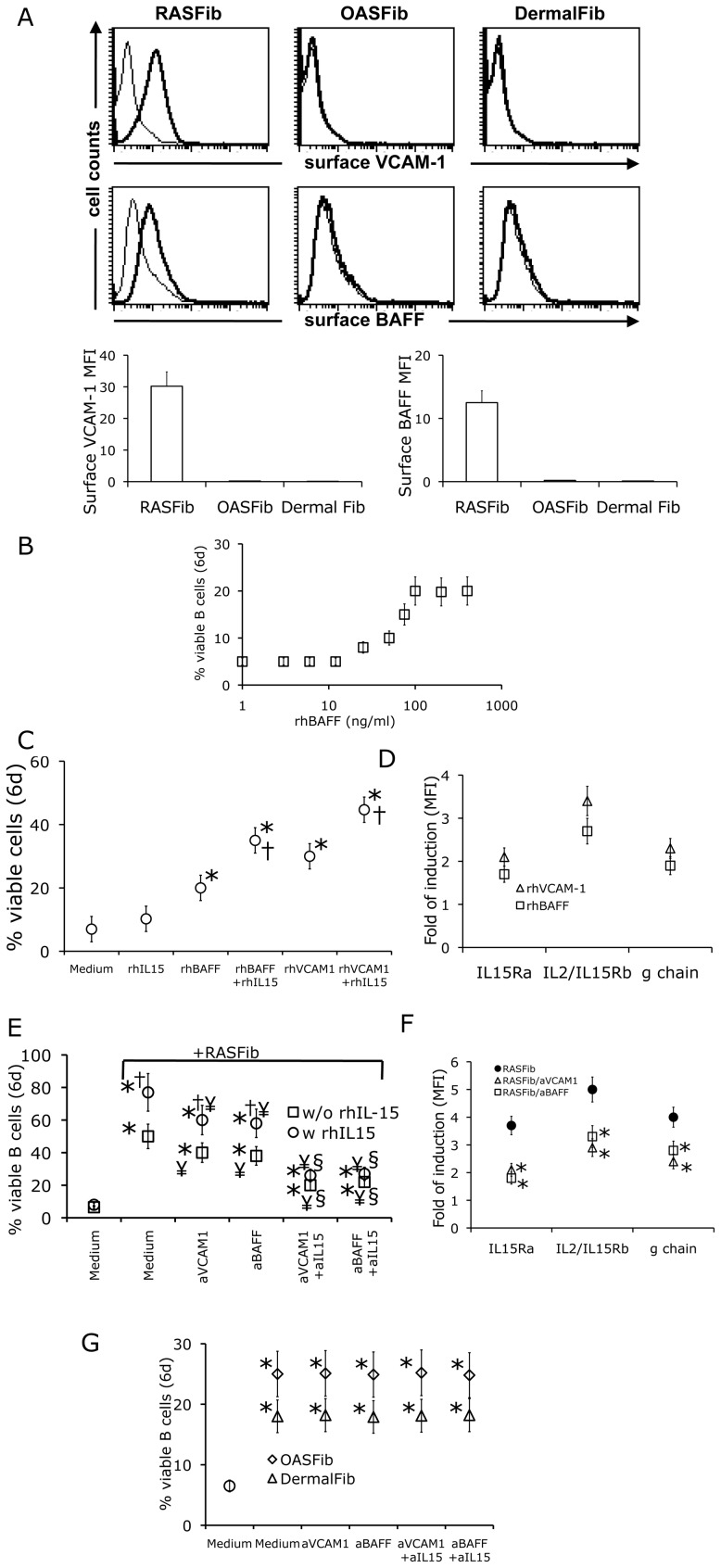
VCAM-1 and BAFF facilitate the effect of IL-15 on B cell survival and upregulate B cell IL-15R expression. **A**. Surface expression of VCAM-1 and BAFF on non-permeabilized RASFib, OASFib or dermal fibroblasts. Top panels: representative flow cytometry histograms. Lower panels: bar histograms showing the mean and SD of the MFI (mean fluorescence intensity) of all tested RASFib cell lines (n = 10), OASFIb lines (n = 10) and dermal fibroblast lines (n = 5) **B**. Effect of rhBAFF (1–400 ng/ml) on isolated B cells cultured alone for 6 days. Each point represents the mean and SD of 15 subjects. **C**. Percentage of viable B cells cultured for 6 days in plain medium, medium with rhIL-15 or rhBAFF, VCAM-1-coated plates, rhIL-15 plus BAFF or rhIL-15 plus VCAM-1. *p<0.05 vs medium; †p<0.05 vs same condition without rhIL-15. **D**. Fold of induction of IL-15Rα, β and γ on B cells cultured for 6 days with rhBAFF or rhVCAM-1, as determined by cytometry and referred to freshly isolated cells. **E**. Percentage of viable B cells cultured for 6 days in medium, or cocultured with RASFib in the absence or presence of anti-VCAM-1 or anti-BAFF antibodies, in combination or not with an anti-IL15 antibody, with or without rhIL-15. *p<0.05 vs B cells cultured in medium; †p<0.05 vs same condition without rh IL-15; ¥p<0.05 vs same condition without anti-VCAM-1 or anti-BAFF antibodies. § p<0.05 vs same condition without anti-IL15. **F.** Fold of induction of IL-15R α, β and γ on B cells cocultured for 6 days with RASFib, with or without anti-VCAM-1 or anti-BAFF antibodies, as determined by cytometry and referred to freshly isolated B cells. *p<0.05 vs B cells cocultured with RASFib without antibodies. Each point represents the mean/SD of 15 subjects. **G**. Effect of VCAM-1 or BAFF neutralizing agents on the survival of B cells cocultured with OASFib or dermal fibroblasts. Each point represents the mean and SD of 15 subjects. * p<0.05 vs B cells cultured alone in plain medium.

Consistent with previous reports [8]–[13], RASFib but not OASFib or dermal fibroblasts, demonstrated a significant constitutive surface expression of the B cell survival factors VCAM-1 and BAFF (Figure 5A). In contrast, suboptimal levels of soluble BAFF were present in cell-free supernantants of RASFib (mean±SD, 38±5 pg/ml). In fact, soluble rhBAFF had no effect on B cells when tested at concentrations below 10 ng/ml (Figure 5B). This observation is consistent with above presented results, showing that supernatants of RASFib/B cell cocultures had no effect on the survival of isolated B cells. Therefore we were next interested in examining the possible role of membrane BAFF and VCAM-1 in the RASFib-mediated upregulation of B cell IL15-R chains, and in facilitating the effect of RASFib IL-15 on B cell survival.

B cells cultured in the presence of high concentrations of exogenous rhBAFF (100 ng/ml) or in the presence of plate-bound rhVCAM-1 demonstrated an improved survival rate at 6 days, when compared with B cells cultured in plain medium (Figure 5C). Interestingly, whereas the effect of isolated rhIL-15 on B cell viability was minimal (Figure 3B, 5C), the increased survival rate of B cells stimulated with rhBAFF or rhVCAM-1 was further augmented in the presence of rhIL-15 (50 ng/ml) (Figure 5C). In parallel, an upregulation of IL-15R chains was observed on B cells cultured in the presence of rhBAFF or of rhVCAM-1 (Figure 5D).

We next observed that the anti-apoptotic effect of RASFib on B cells was significantly reduced in the presence of anti-BAFF or anti-VCAM-1 neutralizing antibodies (Figure 5E). Combination of anti-BAFF or anti-VCAM-1 with anti-IL-15 further decreased the survival of B cells cocultured with RASFib (Figure 5E). In addition, anti-BAFF or anti-VCAM-1 neutralizing antibodies significantly decreased the facilitating effect of RASFib on IL-15 action (Figure 5E). In parallel, neutralization of BAFF or of VCAM-1 significantly reduced the upregulated IL-15R α, β and γ chain expression induced on B cells by coculture with RASFib (Figure 5F). Neutralization of VCAM-1 or BAFF had no effect on the suvival of B cells cocultured with OASFib or dermal fibroblasts (Figure 5G ).

### Memory B Cells from the Peripheral Blood of Early RA Patients Demonstrate Higher Levels of IL-15R Expression Together with Superior Survival Rates in Response to rhIL-15 and in Coculture with RASFib

**Figure 6:**
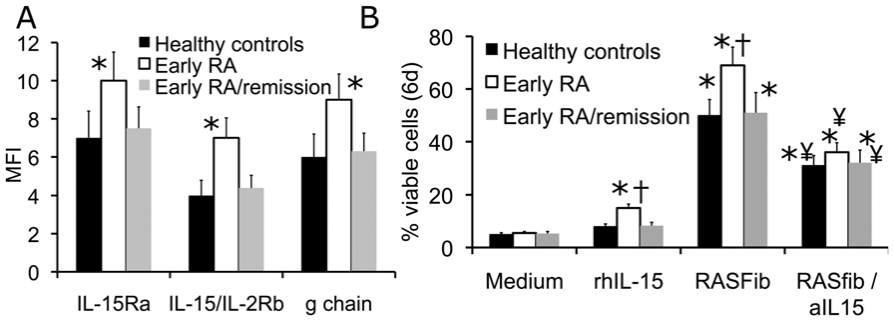
Memory B cells from the peripheral blood of early RA patients demonstrate and upregulated IL-15R expression together with superior survival rates in response to rhIL-15 and in coculture with RASFib. A. Surface expression of IL-15 receptor α, β and γ chains on memory B cells from healthy controls (n = 15) (black bars), from early RA patients (n = 15) (open bars), and from 8 early RA patients who donated blood for a second time after achieving remission (grey bars), as determined by flow cytometry of non-permeabilized cells, and expressed as mean fluorescence intensity (MFI). Each bar represents the mean and SD of 15 or 8 subjects. *p<0.05 vs healthy controls. B. Percentage of viable B cells cultured for 6 days in plain medium, or in medium supplemented with rhIL-15, or cocultured with RASFib in plain medium, or cocultured with RASFib in the presence of a neutralizing anti-IL-15 mAb. Black bars represent B cells from healthy controls (n = 15), open bars represent B cells from early RA patients (n = 15) and grey bars represent B cells from 8 early RA patients who donated blood for a second time after achieving remission. Each bar represents the mean and SD of 15 or of 8 subjects. *p<0.05 vs B cells cultured in plain medium. †p<0.05 vs B cells from healthy controls; ¥p<0.05 vs B cells cocultured with RASFib in the absence of an anti-IL-15 neutralizing antibody.

When compared with memory B cells from the peripheral blood of healthy controls, the MFI of IL-15R α, β and γ chain expression was significantly higher on memory B cells isolated from the peripheral blood of early RA patients (Figure 6A). When stimulated with rhIL-15 in the absence of RASFib, the survival of RA memory B cells was significantly increased as compared with RA memory B cells cultured in plain medium, and opposed to memory B cells from healthy controls which showed a lower response to rhIL-15 (Figure 6B). In B cell/RASFib cocultures, the survival rate of memory B cells from the peripheral blood of early RA patients was significantly higher when compared with healthy controls (Figure 6B). In the presence of IL-15 neutralizing agents, the effect of RASFib on RA memory B cell survival was significantly downregulated. The magnitude of this downmodulation was higher when compared with that observed in memory B cells from healthy controls. Subsequently, in the presence of RASFib and of IL-15 neutralizing agents, no difference was observed between the survival rate of RA memory B cells and the survival rate of memory B cells from healthy controls, suggesting that IL-15 plays an important role in the superior survival rate of cocultured RA memory B cells.

Interestingly, when memory B cells from 8 of the 15 early RA patients were re-examined in a follow-up visit, after remission had been achieved with treatment (oral methotrexate with or without low-dose prednisone) (Table 2), their IL-15R expression and their behaviour in coculture with RASFib were no longer different from healthy controls (Figure 6A, B).

## Discussion

We have herein shown for the first time that IL-15 expression on RASFib significantly contributes, through a cell-contact dependent mechanism, to the previously described anti-apoptotic effect of RASFib on B cells [6]–[11].

IL-15, initially described as a T cell growth factor [19], [28], has been reported to costimulate the proliferation and differentiation of activated B cells [21]. IL-15 acts through a heterotrimeric receptor consisting of a specific high-affinity binding α-chain (IL-15Rα) plus the IL-2 receptor subunits β- and common γ-chain that mediate signalling [29], [30]. The high affinity of IL-15Rα conditions an extremely rapid uptake of secreted IL-15, thereby preventing detection of IL-15 in culture supernatants [31]. Subsequently, most of the IL-15 detected on cell surfaces is bound to IL-15Rα [31] and can stimulate in *trans* both βγ- and IL-15R-αβγ- bearing cells [31]. The presence of surface IL-15Rα-bound IL-15 is synonimous of active IL-15 secretion, and the level of expression of surface IL-15 in a given cell population may reflect the rate of internalization of the IL-15/IL-15Rα complex. Thus, in contrast with IL-2, IL-15 can be expressed on the cell surface, where it is able to exert biological functions through cell contact-dependent mechanisms [29]–[31]. IL-15 is expressed intracellularly by monocyte-macrophages [29], [30], dendritic cells [29], [30] and fibroblasts [30], [32]. Surface IL-15 is constitutively present and physiologically active on follicular dendritic cells (FDCs) [22] and on certain but not all fibroblast lineages [16]. IL-15 on FDCs has been described to promote B cell survival and proliferation [22]. IL-15 on fibroblasts from human spleen regulates NK cell differentiation from blood CD34+ progenitors [33] and IL-15 on bone marrow fibroblast-like stromal cells contributes to T cell recruitment and expansion in aplastic anemia [34]. We previously reported that constitutively expressed IL-15 on the surface of RASFib induces T cell activation and cytokine secretion through direct cell contact [16] and is also able to modulate the equilibrium between regulatory and responder CD4 T cells [17]; in contrast, resting OASFib or dermal fibroblasts do not modulate T cell biology, consistent with their lack of constitutive surface IL-15 expression [16], [17].

Our initial hypothesis stated that RASFib surface IL-15 plays an important role in promoting contact-dependent survival of B cells. This was confirmed in experiments with neutralizing antibodies to IL-15, IL-15Rα and with a soluble IL15Rα-Fc chimera. Interestingly, whereas the effect of RASFib on B cell survival and differentiation was downmodulated by IL-15 neutralizing agents and upregulated by rhIL-15, rhIL-15 itself did not significantly modify the survival of isolated peripheral blood B cells from healthy controls. This is in agreement with previously reported results indicating that resting B cells are not responsive to exogenous rhIL-15 in the absence of a facilitating stimulus [21].

Indeed, we observed that RASFib themselves seemed to facilitate the response to IL-15 through an upregulation of B cell IL-15R chains that was mediated by initially unidentified factors. Subsequent experiments indicated that BAFF and VCAM-1, two B cell survival molecules expressed on the surface of RASFib, are able to mediate IL-15R upregulation. This is consistent with published reports describing a facilitating effect of VCAM-1 and of BAFF on the function of B cell surface molecules [35], [36] and indicates that cooperation among various surface molecules is responsible for the effect of RASFib on B cell survival. In fact, observations from other authors describing the action of RASFib on B cells, had noted that functional blocking of BAFF or of VCAM-1 had a partial effect on downregulating B cell survival [8]–[11], which suggested the contribution of additional B cell trophic factors. We also observed that OASFib and dermal fibroblasts, which do not constitutively express IL-15, VCAM-1 nor BAFF on the cell membrane, were significantly less effective than RASFib at promoting B cell survival, and neutralization of these agents had no effect on the survival of cocultured B cells.

Importantly, experiments with transwell inserts and with coculture supernatants indicated that soluble factors are not sufficient to prolong B cell survival in our system, which is in agreement with published observations [9]. Soluble IL-15 was not detected in coculture supernatants; in addition, very low amounts of soluble BAFF were present, and exogenous rhBAFF could not halt B cell apoptosis at concentrations below 10 ng/ml, consistent with previous work supporting the necessity of direct cell contact for the effect of BAFF expressed on RASFib to take place [12], [13].Peripheral blood B cells from RA patients have been shown to display several specific features [37]–[39] indicative of a preactivated state. We observed for the first time an upregulated expression of IL-15R α, β and γ chains on memory B cells from the peripheral blood of early RA patients. This resulted in an enhanced response to rhIL-15 and to RASFib IL-15, with further improved survival rates when cocultured ex vivo with RASFib. Interestingly, when memory B cells from 8 of the 15 early RA patients were re-examined in a follow-up visit, after remission had been achieved with treatment (oral methotrexate with or without low-dose prednisone), their IL-15R expression and their behaviour in coculture with RASFib were no longer different from healthy controls.

### Conclusions

In summary, the results presented herein indicate that RASFib IL-15 expression may be an important contributor to the initiation and perpetuation of the inflammatory process in RA. Under physiological conditions, B cells continuously traffick through lymphoid and non-lymphoid organs [40], [41], and B cell persistence in tissues is conditioned by local stromal factors [42]. The constitutive IL-15 expression on the surface or RASFib, cooperating with VCAM-1 and BAFF, is likely to create a specific microenvironment promoting B cell fitness. This may provide a survival niche favoring the persistence of B cells in the synovium despite intensive therapy with biologicals such as anti-TNF and anti-CD20 mAbs.
